# Machine learning to guide clinical decision-making in abdominal surgery—a systematic literature review

**DOI:** 10.1007/s00423-021-02348-w

**Published:** 2021-10-29

**Authors:** Jonas Henn, Andreas Buness, Matthias Schmid, Jörg C. Kalff, Hanno Matthaei

**Affiliations:** 1 Department of General, Visceral, Thoracic and Vascular Surgery, University of Bonn, Bonn, Germany; 2 Institute for Medical Biometry, Informatics and Epidemiology, University of Bonn, Bonn, Germany; 3 Institute for Genomic Statistics and Bioinformatics, University of Bonn, Bonn, Germany

**Keywords:** Abdominal surgery, Machine learning, Clinical decision-making, Risk prediction, Postoperative complications, Digitalization

## Abstract

**Purpose:**

An indication for surgical therapy includes balancing benefits against risk, which remains a key task in all surgical disciplines. Decisions are oftentimes based on clinical experience while guidelines lack evidence-based background. Various medical fields capitalized the application of machine learning (ML), and preliminary research suggests promising implications in surgeons’ workflow. Hence, we evaluated ML’s contemporary and possible future role in clinical decision-making (CDM) focusing on abdominal surgery.

**Methods:**

Using the PICO framework, relevant keywords and research questions were identified. Following the PRISMA guidelines, a systemic search strategy in the PubMed database was conducted. Results were filtered by distinct criteria and selected articles were manually full text reviewed.

**Results:**

Literature review revealed 4,396 articles, of which 47 matched the search criteria. The mean number of patients included was 55,843. A total of eight distinct ML techniques were evaluated whereas AUROC was applied by most authors for comparing ML predictions vs. conventional CDM routines. Most authors (*N* = 30/47, 63.8%) stated ML’s superiority in the prediction of benefits and risks of surgery. The identification of highly relevant parameters to be integrated into algorithms allowing a more precise prognosis was emphasized as the main advantage of ML in CDM.

**Conclusions:**

A potential value of ML for surgical decision-making was demonstrated in several scientific articles. However, the low number of publications with only few collaborative studies between surgeons and computer scientists underpins the early phase of this highly promising field. Interdisciplinary research initiatives combining existing clinical datasets and emerging techniques of data processing may likely improve CDM in abdominal surgery in the future.

## Introduction

Abdominal surgery is associated with the risk for severe morbidity and mortality, which is why clinical decision-making (CDM), and particularly the indication for an operation, remains a critical task of all surgical disciplines [1]. Here, a potential imbalance between risks and benefits needs to be avoided by processing and interpreting perioperative data to improve CDM. Treatment guidelines for virtually any diagnosis were created to utilize this vastly available data consisting of medical history, radiologic data, and molecular data to determine the need (benefit of) for surgery [2]. However, these oftentimes provide consensus-level recommendations rather than statistical evidence, which is why surgeon and patient are left with uncertainty regarding a procedures benefit [3]. Furthermore, various risk scores have been established to support CDM by minimizing the human error source using statistical evidence in their model [4, 5]. Yet, such scores lack the option to properly adapt to individual medical histories since their statistical assumptions are quite general. Additionally, larger prospective studies supporting the scores’ performance are scarce [6]. In conclusion, neither benefits nor risks can yet be evaluated on an individual and higher evidence-based level.

National registries, like the Study, Documentation and Quality Center (StuDoQ) of the German Association for General and Visceral Surgery (DGAV), aimed at supporting quality management of surgical therapy by collecting high-quality perioperative data maintained in a standardized prospective multicenter fashion. Such databases showed excellence performance in assessing the uses and risks of operations and therefore represent a foundation for innovative approaches of data analyses [7]. Growth of medical data collections is additionally facilitated by modern tools of automated data mining (e.g., natural language processing), which is why adequate analysis is rendered even more laborious [8]. There are numerous examples of successful applications of modern computational tools for data interpretation in modern medicine with spectacular advances (i.e., pathology and radiology) [9, 10]. For example, supervised machine learning (ML), as a subdomain of artificial intelligence (AI), intends to learn classification rules based on given examples. In detail, supervised learning uses annotated data (i.e., known predictor and outcome variables from retrospective cases) to calculate predictions for unknown cases given the values of the predictor variables [11]. The combination and integration of both datasets and modern data science techniques are attributed to a possibility to revolutionize CDM in surgery [12]. Extensive national and international research programs (e.g., National Strategy for Artificial Intelligence, Federal Ministry of Education and Research, Germany, or the Coordinated Plan on Artificial Intelligence of the European Union) highlight the political support and appreciated significance of AI and the opportunity of a successful implementation. With existing uncertainties in surgical CDM, there is an urge to assess the potential power of the recently defined field of surgical data science for improved decision support in patient care [12]. To provide an accurate overview of ML in CDM, we present a systematic review of the literature with focus on abdominal surgery.

## Methods

### Identification and selection of studies

**Fig. 1:**
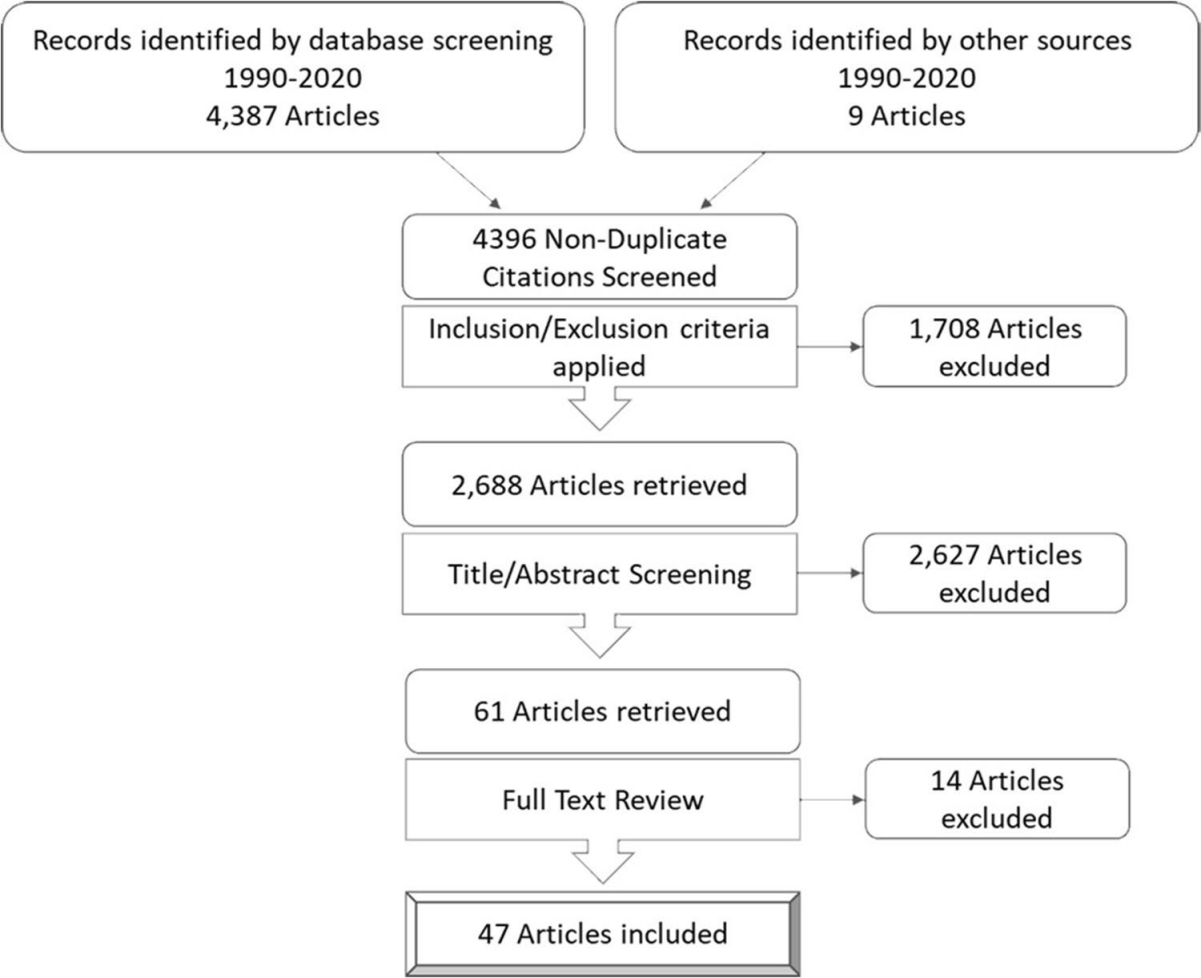
PRISMA flowchart for selecting relevant publications. All nine citations from other sources were found in references of finally included publications

We performed a systematic literature search to assess the evidence of ML’s use for CDM in abdominal surgery. To establish a relevant query, the *PICO* framework was applied [13]. Insufficient evidence in CDM in abdominal surgery depicts the addressed problem. We aimed to evaluate ML’s use as intervention and compared it to conventional decision-making. Outcome of interest was a more precise determination of either benefits or risks of abdominal operations for a subsequently more personalized CDM. Assessed risks included mortality and morbidity and benefits were assumed if a desired effect of a given operation (i.e., cancer survival, cure of disease, positive effect of surgery) was given. A distinct search algorithm was applied using the PubMed database, whereas the search was guided by *The PRISMA Statement* for systematic reviews [14]. The query was conducted January 2021 by inserting the keywords “*surgery machine learning*” into PubMed. Each article was processed using a standardized procedure: We considered articles between 1^st^ of January 1990 and 31^st^ of December 2020 that were published in peer-reviewed journals in the English language. Reviews, comments, and any other articles representing no original research were excluded. Articles were then screened for their contribution to CDM in abdominal surgery, whereas only articles that aimed for assessment of perioperative risk or benefits for surgery were included. At first, titles were analyzed and in case of interest associated abstracts were extracted and examined. Secondly, full-text review was undertaken whenever the abstract fulfilled our criteria and addressed the search question. References of every article included were scrutinized for additional research studies of interest. Figure 1 shows the PRISMA flow diagram of our query.

### Data extraction and analysis

Subsequently, a qualitative and quantitative analysis of the included articles was conducted. Full-text review was performed as defined within the PICO Framework. Hence, all selected articles were examined for journal topic, surgical domain, number and composition of cohorts, study timing, whether it was conducted retro- or prospectively, outcome focused on, ML technique applied, number of included predictor variables, method to compare ML with, results of comparison, strengths, and limitations, and finally predicted impact on CDM. If applicable, reported AUROC values with 95% confidence intervals were retrieved for ML and compared conventional technique. To allow for overall better analysis, the best performing ML and conventional technique were used. Analyses were conducted in Microsoft Excel, Version 2102 (Microsoft, Baltimore, USA); R (R Foundation for Statistical Computing, Vienna, Austria); and RStudio version 1.3.1093 (RStudio, Inc., Boston, USA).

## Results

### Study characteristics and design

**Fig. 2:**
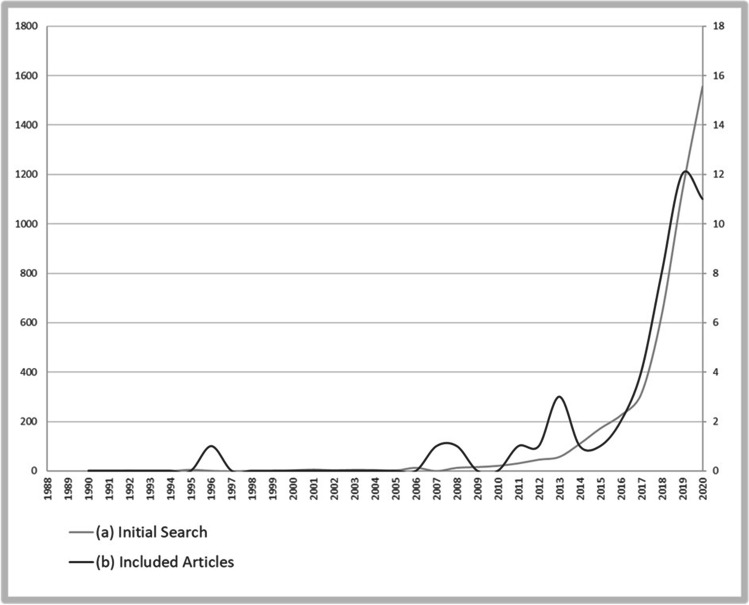
Number of articles (a) retrieved by unfiltered search query and (b) eventually included in the review. Years are displayed on the *x*-axis, whereas number (a) is shown on the left *y*-axis and (b) on the right *y*-axis

Our search resulted in 4,396 records, of which a total of 47 articles were included in the final literature review process. A large fraction of articles (*N* = 1,708) was excluded for non-English language or lack of original research. Furthermore, 2,627 records were excluded because they were not addressing topics in abdominal surgery (e.g., neuro-, cardiothoracic-, trauma-, orthopedic-, and ENT-surgery). After full-text review, fourteen articles were excluded since articles did not investigate the assessment of risks or benefits of surgery. From 1990 until today, the number of studies regarding ML in abdominal surgery has increased with significant rise in the past decade (see Fig. 2). Articles were mainly published in journals of the following medical areas: surgery (*N* = 19, 40.4%), internal medicine (*N* = 8, 17.0%), bioinformatics (*N* = 8, 17.0%), anesthesia (*N* = 3, 6.4%), and others (*N* = 9, 19.1%). To provide an overview of encompassed fields of diagnosis, those publications were grouped into the following clinical domains: general surgery (*N* = 13, 27.7%), colorectal surgery (*N* = 7, 14.9%), liver transplantation (*N* = 6, 12.8%), acute appendicitis (*N* = 5, 10.6%), bariatric surgery (*N* = 4, 8.5%), pancreatic surgery (*N* = 4, 8.5%), hepatic surgery (*N* = 3, 6.4%), emergency surgery (*N* = 2, 4.3%), oncologic surgery (*N* = 2, 4.3%), and esophagus surgery (*N* = 1, 2.1%). In Table 1, an overview of included research articles is provided. The mean patient number was 55,842.5 (SD, 167,592.3; median, 1003.0; IQR 377.0–47,189.5). Mean period of research was 95.5 months (SD, 66.8; median, 82.5; IQR, 49.3–130.0). With exception of one prospective study [15], all other research was conducted in a retrospective fashion. Studies either focused on predicting the risk (*N* = 26, 55.3%) or the benefit (*N* = 21, 44.7%) of procedures.

**Table 1:** Study characteristics

Reference		Surgical domain	Predicted outcome	Outcome variable	Patients	Study period (m)	ML	Predictor variables	Cross-validation	Benchmark	∆AUROC
Benefit											
Andres [16]	LT		OS	Death	2769	142	Other	17	Yes	NA	NA
Ansari [17]	Pancreatic		OS	Death	84	188	ANN	33	Yes	Cox	NA
Aron-Wisnewsky [18]	Bariatric		DM remission	Treatment needed	352	132	SVM	NA	Yes	Scores	0.06
Briceño [19]	LT		Graft survival	Graft mortality	1003	23	ANN	57	Yes	Scores	0.13
Cruz-Ramírez [20]	LT		Graft survival	Graft mortality	1003	23	ANN	64	NA	NA	NA
Debédat [21]	Bariatric		DM remission	Treatment needed	175	132	SVM	NA	NA	Scores	0.09
Ho [22]	Hepatic		DFS	Death/recurrence	427	84	ANN	31	NA	LR	0.01
Hsieh [23]	Appendicitis		Diagnosis	Histopathology	180	35	RF	16	Yes	LR	0.11
Ichimasa [24]	Colorectal		Diagnosis	Metastasis	690	179	SVM	45	NA	LR	0.02
Johnston [25]	Bariatric		DM remission	Treatment needed	16,527	81	Other	125	Yes	NA	NA
Kuwahara [26]	Pancreatic		Diagnosis	Carcinoma	206	267	ANN	11	Yes	LR	0.25
Lau [27]	LT		Graft survival	Graft mortality	180	64	RF	173	NA	Scores	0.16
Maubert [28]	Oncologic		Respectability	Operation performed	763	191	RF	9	NA	NA	NA
Pesonen [29]	Appendicitis		Diagnosis	Histopathology	911	84	ANN	43	NA	NA	NA
Prabhudesai [30]	Appendicitis		Diagnosis	Histopathology	60	6	ANN	11	NA	NA	NA
Rahman [31]	Esophagus		DFS	Death/recurrence	812	156	GB	11	Yes	NA	NA
Reismann [32]	Appendicitis		Diagnosis	Histopathology	590	117	Other	10	NA	Scores	0.05
Sakai [33]	Appendicitis		Diagnosis	Histopathology	169	144	ANN	9	Yes	LR	0.02
Springer [34]	Pancreatic		Diagnosis	Carcinoma	862	49	Other	NA	NA	Scores	NA
Tsilimigras [35]	Hepatic		OS	Death	1146	335	RF	20	Yes	NA	NA
Xu [36]	Colorectal		DFS	Death/recurrence	999	120	GB	18	NA	LR	0.07
Risk											
Bertsimas [37]		Emergency	Mortality	30d death	382,960	84	RF	150	NA	Scores	0.02
Bihorac [38]		General	Mortality	30d death	51,457	130	RF	285	Yes	NA	NA
Brennan [15]		General	Mortality	30d death	150	130	RF	285	NA	Experts	0.26
Bronsert [39]		General	Morbidity	Any complication	6840	40	ANN	838	Yes	NA	NA
Cao [40]		Bariatric	Morbidity	complication	44,061	60	ANN	16	Yes	LR	0.03
Cao [40]		Emergency	Mortality	90d death	157	24	RF	25	Yes	LR	0.05
Chen [41]		Colorectal	Morbidity	Bleeding	12,402	192	GB	117	Yes	LR	0.09
Chiew [42]		General	Mortality	30d death	90,785	57	RF	26	Yes	Scores	0.07
Chiu [43]		Hepatic	Mortality	1y death	434	NA	ANN	33	NA	LR	0.08
Corey [44]		General	Mortality	30d death	99,755	60	RF	194	Yes	Scores	0.12
Datta [45]		General	Mortality	Inhouse death	43,943	57	RF	367*	Yes	NA	NA
Ehlers [46]		General	Mortality	90d death	410,521	60	BN	300	NA	Scores	0.19
Ershoff [47]		LT	Mortality	90d death	57,544	120	ANN	202	Yes	Scores	0.02
Francis [48]		Colorectal	Morbidity	Stay > 7d	275	84	ANN	16*	NA	LR	0.01
Fritz [49]		General	Mortality	30d death	95,907	50	ANN	56*	NA	LR	0.03
Hill [50]		General	Mortality	Inhouse death	52,894	68	RF	58	Yes	Scores	0.07
Hyer [51]		General	Morbidity	Any complication	1,049,160	24	Other	NA	NA	Scores	0.07
Jauk [52]		General	Morbidity	ICU admission	61,864	98	RF	630	Yes	NA	NA
Kambakamba [53]		Pancreatic	Morbidity	Pancreatic fistula	110	60	RF	NA	Yes	Experts	0.10
Lee [54]		General	Mortality	Inhouse death	59,985	39	ANN	87*	Yes	LR	0.01
Liu [55]		LT	Mortality	30d death	480	120	RF	13	Yes	LR	0.10
Merath [56]		Oncologic	Morbidity	Any complication	15,657	24	ANN	34	NA	Scores	0.03
Soguero-Ruiz [57]		Colorectal	Morbidity	Anastomotic leakage	402	72	SVM	9	Yes	NA	NA
Sohn [58]		Colorectal	Morbidity	SSI	1856	24	BN	31	Yes	LR	0.11
Thottakkara [59]		General	Morbidity	Sepsis	50,318	130	BN	285	Yes	LR	-0.02
Weller [60]		Colorectal	Morbidity	Bleeding	4773	36	RF	NA	NA	NA	NA

*LT* liver transplantation, *OS* overall survival, *DM* diabetes mellitus, *DFS* disease-free survival, *ICU* intensive care unit, *ML* machine learning technique used for analysis, *ANN* artificial neural network, *SVM* support vector machine, *RF* random forest, *GB* gradient boosting, *BN* bayesian network, *NA* not available/not applicable, *Cox* cox regression, *LR* logistic regression, *AUROC* area under the receiver operating characteristic

^*^These studies additionally incorporated intraoperative predictor variables

### Technical approaches

Conventional measures of CDM were represented by various scores and tests, including logistic regression (*N* = 16, 34.0%), specific scores (*N* = 14, 29.8%), expert opinion (*N* = 2, 4.3%), and Cox regression (*N* = 1, 2.1%). The remaining articles (*N* = 14, 29.8%) did not perform statistical comparison. Specific scores comprised ASA classification, ACS NSQIP Surgical Risk, Charlson comorbidity index, DiaRem, Donor Risk Index for Liver Transplantation, Elixhauser comorbidity index, Model for End-stage Liver Disease (MELD), appendiceal diameter, and survival outcomes following liver transplantation (SOFT). Authors held insufficient precision (*N* = 26, 55.3%), the predictors linearity (*N* = 5, 10.6%), missing automation (*N* = 5, 10.6%), and subjectiveness (*N* = 2, 4.3%) responsible for conventional CDM’ insufficiency, while nine authors (19.1%) did not specify. There were eight common ML techniques applied: artificial neural network (*N* = 16, 34.0%), random forest (*N* = 16, 34.0%), support vector machine (*N* = 4, 8.5%), gradient boosting (*N* = 3, 6.4%), and Bayesian network (*N* = 2, 4.3%). Five studies (10.6%) used individually constructed and named algorithms. Also, some articles made use of natural language processing to extract data. Furthermore, the outline of every ML method used varied among the publications ranging from detailed technical workflows in the “Methods” section to a simple statement which algorithm was used. The mean number of predictor variables integrated in ML algorithms was 116.1 (SD, 171.8; median, 34.0; IQR 16.0–150.0). All studies relied on preoperative predictor variables, while 4 (8.5%) studies additionally included intraoperative data. Over two-thirds of included studies (*N* = 32, 68.1%) emphasized the importance of variable selection when designing ML approaches. Many authors (*N* = 27, 57.4%) used internal cross-validation, of which three additionally used external validation [18, 25, 31].

### Primary outcome

Most studies (*N* = 41, 87.2%) used the receiver operating characteristic curve (ROC) to contrast the true positive rate against the false positive rate. Then, the area under the ROC curve (AUC) was calculated, resulting in AUROC values. The remaining six studies (12.8%) either used other or no measures to display their results. The mean AUROC for ML techniques in the observed articles was 0.84 (SD, 0.10; median, 0.84; IQR, 0.78–0.91). In contrast, the chosen benchmarks (i.e., conventional techniques) reached a mean AUROC of 0.76 (SD, 0.11; median, 0.77; IQR, 0.69–0.86), resulting in a mean difference of 0.08 (SD, 0.07; median, 0.07; IQR, 0.03–0.10). Herein, all but one study stated ML’s superiority over the chosen benchmark (see Table 1).

### Considerable aspect

In addition to ML’s performance, every third (*N* = 16, 34.0%) article concluded that ML will strongly enhance personalized medicine. Furthermore, many authors (*N* = 12, 25.5%) elaborated that ML can spare the already scarce monetary resources in healthcare systems. While improved allocation was mostly (*N* = 9/12, 75.0%) held accountable, remaining authors (*N* = 3/12, 25.0%) stressed the low cost of ML techniques. However, only three articles in detail explicated how the application of ML might save healthcare costs. Nearly half (*N* = 19, 40.4%) of all studies distinctively address the surgeons (physicians) role when using ML for CDM. Of those, most authors discussed support (*N* = 11/19, 57.9%) and guidance (*N* = 6/19, 31.6%) by ML for clinicians, whereas one study highlighted the physician’s role in implementing ML into CDM.

### Risks and benefits of surgery

Risk stratification of surgery itself was mostly addressed by large population-driven studies (mean number of patients, 99,795.8; SD, 215,498.9; median 44,002.0; IQR, 824.0–61,394.3). An average number of 176.4 predictor variables were included into the trained ML models (SD, 207.0; median, 87.0; IQR, 28.5–285.0). Patients and their outcome were followed over a mean time of 73.7 months (SD, 42.0; median 60.0; IQR, 40.0–98.0). In detail, those studies demonstrated that ML could outperform conventional CDM in precisely predicting risk for adverse events after surgical intervention. For example, Chiew et al. used a set of 90,785 patients for precise prediction of postoperative mortality. They furthermore concluded that ML techniques can include more clinical features than conventional CDM and even have the possibility for real-time updates once new crucial features are identified [42]. Additionally, Fritz et al. anticipated that ML may help clinicians to identify patients with particularly lethal risk with the chance to adapt their clinical decisions to this hazard [49]. Likewise, Bihorac et al. successfully used records from 51,457 patients to test ML in predicting complications, with exciting results [38]. Subsequently, the same group prospectively tested their innovative ML application against conventional “clinical judgement” and demonstrated that their ML algorithm outperformed the clinical experts [15]. Furthermore, this review unveiled reasonable evidence for improvement of perioperative care through ML. Specifically, two studies discussed the use of ML in the prediction of need for intensive care resources, stating that better allocation will improve individual treatment [42, 52]. Despite these obvious advantages of large cohorts, disease-specific questions, especially assessment of benefits of surgery, are mainly tackled by well-curated datasets for an exactly defined clinical scenario (mean number of patients, 1424.2; SD, 3427.2; median, 690.0; IQR, 180.0–999.0). In general, those studies included less predictor variables (mean, 39.1; SD, 43.0; median, 19.0; IQR, 11.0–44.5) but included data from larger time spans (mean months, 121.5; SD, 80.2; median, 120.0; IQR, 64.0–156.0). For instance, Hsieh et al. were able to facilitate a random forest model to succeed other scores in the safe diagnosis of acute appendicitis, proving that ML is a useful tool to evaluate patients in need for surgery [23]. In an oncological setting, Ichimasa et al. focused on patients who underwent endoscopic resection for T1 colorectal cancer and evaluated the use of ML in predicting if patients suffered from simultaneous lymph node metastasis. In consequence, patients identified through this approach would be referred to additional surgical resection for improved outcome. Thus, the group successfully demonstrated that there is a realistic chance of reducing unnecessary operations [24]. Furthermore, Springer et al. charged a comprehensive test with molecular data from pancreatic cysts and clinical features and were able to identify patients more adequately in need for pancreatic surgery [34]. Finally, Johnston et al. implemented ML to predict the need of anti-hyperglycemic medication after laparoscopic metabolic surgery and their model showed promising results in enhanced patient selection [25].

### Limitations

While most authors did outline specific limitations to their studies (*N* = 37, 78.7%), none was specified in ten publications (21.3%). Limitations were grouped into insufficient data (*N* = 20), structural weaknesses (*N* = 19), selection bias (*N* = 9), and problems with interpretability (*N* = 7). Structural weaknesses included a lack of external validation and single-center approach. Of note, no differences between larger (risk stratification) studies and smaller (benefit assessment) ones were observed for interpretability, structural weaknesses, or selection bias. However, studies with larger patient cohorts for risk stratification more often mentioned problems with insufficient data. Eventually, most studies (*N* = 29, 61.7%) outlined the need for proper evaluation by extended research. Additionally, the so-called *black box* phenomenon was repeatedly stated: some ML techniques use algorithms which make the understanding of the connection between factors and predicted outcome demanding. In addition to resulting interpretability concerns, the black box hinders detection of yet unknown possible causalities.

## Discussion

In operative medicine, oncological and emergency surgery are disciplines where rapid and vitally important decisions are needed. Yet, currently available mechanisms (i.e., treatment guidelines and scores) are insufficient in including existing data for suited strategies [34, 42]. Additionally, growing datasets that need exploration for possible use are expanding rapidly and automatically [8]. This incomplete use of already existing and newly available data is unacceptable when human lives are at stake. Thus, evaluation of modern techniques (i.e., ML) is imperatively needed to close this gap [12]. Fortunately, surgeons, anesthesiologists, and data analysis experts seem equally interested in the use of ML for surgical CDM, as reflected by journals in which the articles were published. For future research, collaboration work of those disciplines is urgently desired to guarantee improved outcome. Moreover, the growing relevance of ML in surgical CDM is reflected by the increasing number of studies published recently while this interdisciplinary collaborative field is still in its infancy. Even at this infant level, presented results show that ML is at least comparable, if not superior to conventional CDM mechanisms.

In detail, studies with mostly smaller sample sizes already show ML’s capability for a more personalized approach in surgical indication. Refined datasets can, even for rare conditions, pool worldwide accessible data to facilitate a comprehensive algorithm to counsel patients and caretakers regarding the need for surgery. For example, residents in the emergency room need to make decision under unfavorable conditions (e.g., night shift). Although an algorithm predicting the need for emergency surgery cannot replace structured diagnosis and consulting a more experienced physician, it might help selecting patients in need for dedicated attention. Moreover, multidisciplinary tumor boards discussing treatment plan for cancer patients could profit from ML counseling for a more individualized therapy. On the other hand, large population-driven algorithms can be used for precise and individualized risk assessment. In a first step, digital assistants (e.g., smartphone app or IT system plugins) could analyze patient and hospital sited predictor variables to allow for a best-informed decision for both patients and surgeons [38]. Once settled for an operation, surgeons and anesthesiologists could profit from the risk assessment for enhanced resource allocation.

Monetary concerns are growing in our commercialized healthcare systems and the so-called super users have been identified as a lucrative target for cost reduction. Identifying (aka hot spotting) super users, who have an increased demand for resources after surgery, is a known cost-containment strategy. Here, Hyer et al. demonstrated the effective use of ML for improved hot spotting [51]. Moreover, ML is capable of further containing cost by its initial low costs as well as the ability to enhance (monetary) resource allocation by targeting patient at risk with distinct prehabilitation measures and dedicated perioperative care [25, 41]. However, the true effect is yet unknown and needs meticulous evaluation by future studies. Herein, carefully assessing the interaction between algorithms and surgeons (physicians) plays a central role in lifting ML approaches from digital bench to bedside [15]. Currently, authors recognized the elimination of subjectiveness and “eminence based” influences in CDM, resulting in more data-driven and evidence-based predictions. However, the need for continuous supervision of ML applications by surgeons is of sincere concern because evidence of ML’s superiority is still on an investigational level. One of the central ethical questions remains if technology (i.e., ML) might replace human doctors and the accompanying human relationship between patient and physician [50]. On the other hand, interdisciplinary teams already make use of statistical and mathematical models (i.e., guidelines for cancer treatment relying on staging). So why not make complementary use of ML to, for example, reduce unnecessary operations [24]? Thus, surgeons must embrace algorithms as an additional tool in their portfolio rather than a menace to their integrity. Accordingly, most authors see ML as a complementary tool for CDM, rather than a replacement for human experience. This is in accordance with *Eric Topol’s* view on the confluence of human and AI, who concluded that human health is too precious for eliminating doctors completely from the process of diagnosis and therapeutic counseling [61].

The first step for future research approaches in ML must comprise a definite research question for following adequate methodical considerations. Before developing a tailored algorithm, researchers must identify a suitable dataset for the desires task. In principle, larger cohorts can improve statistical power and thus are preferably used. They come, however, with the tendency of not being sufficiently tailored to the clinical population of interest. Especially annotation of data (i.e., making the data usable for the machine) is an important factor for successful algorithms, but is limited by time-consuming human work [12]. Specialized multicenter registries have proven to effectively pool clinical data in rare scenarios, which is why they might be one cornerstone in supplying large-scale high-quality data for successfully implementing ML in surgical CDM [12, 62]. Additionally, automated data annotation needs to get more evaluation for a maximized facilitation of larger data volumes [12]. Once the dataset is chosen, bias and confounders must be carefully assessed and delicately targeted, although they never can be eliminated [63]. Next, an appropriate ML algorithm and its’ suited benchmark must be chosen. Mainly comparison with experts and widely used statistical models (i.e., logistic regression) bring the chance of studying ML’s true power for real-life applications [64]. Furthermore, the underlying creational process must be detailedly outlined to allow for transparent reading. In detail, selecting appropriate predictor variables to include into an algorithm is crucial to guarantee successful models [40]. Eventually, for reporting results, AUROC seems the most established tool for model evaluation. However, most medical applications have skewed datasets since diseases or adverse events depict the minority of observed cases. For example, false-negative predictions are the worst case for patients and caretakers in an oncological setting, but the needed sensitivity is not fully represented by AUROC. In contrast, precision-based metrics like AUPRC demonstrate an algorithms’ weakness to imbalanced datasets, thus giving additional crucial information [42, 45]. Additionally, it is usually of interest to evaluate the accuracy of predicted risk probabilities by model calibration [65]. In conclusion, the use of single performance measures is insufficient, which is why future studies must include multiple tools and compare their individual strengths and weaknesses [66].

Our review has relevant limitations: Firstly, the vast heterogeneity of selected studies regarding ML techniques, cohort composition, and surgical disciplines renders comparison difficult on some levels. Therefore, technical accuracy was sacrificed in favor of a more comprehensive overview of ML in abdominal surgery and a statistical meta-analysis could not reasonably be conducted. Secondly, by setting search criteria a priori to guarantee objectivity, a complete representation of all relevant work cannot be achieved. In detail, database searches may leave relevant articles concealed because they possibly did not use certain keywords. The selection of articles might be further influenced by the manual full text review, which cannot fully exclude subjective factors. Finally, as for any review, our results in this rapidly emerging field are most likely outdated with the day of data acquisition. Yet, the retrospective contemplation of research can identify research trends and generate an appropriate outlook.

## Conclusion

ML has irreversibly found its way in our daily life and into CDM in medicine, while the existing evidence merely allows a first glance at this innovative approach. Even though huge datasets already exist, and ML has become an established technique in the medical field, there is only preliminary work to integrate both in surgical decision-making. Reviewed data rather allow for a first estimation of ML’s power and possibilities, whereas ML appears to outperform conventional CDM. Improving precision of predicting benefits as well as risks holds the opportunity to revolutionize CDM in abdominal surgery. While from the current standpoint an entire replacement of humans in CDM is unrealistic with respect to technical and ethical reason, surgeons should start integrating ML and other new technologies into their clinical routines. Thus, it is our imperative task to support the ongoing digitalization in respect of CDM in abdominal surgery by collaborative research with computer scientist for an optimized patient outcome.

## Data Availability

The data that support the findings of this study are available from the corresponding author, HM, upon reasonable request.
